# Seroprevalence of SARS-CoV-2 IgG among healthcare workers in Lagos, Nigeria

**DOI:** 10.1371/journal.pone.0292440

**Published:** 2023-10-05

**Authors:** Fehintola Anthonia Ige, Gregory Aigbe Ohihoin, Kazeem Osuolale, Adedamola Dada, Ngozi Onyia, Adeyemi Johnson, Azuka Patrick Okwuraiwe, Omoladun Odediran, Gideon Liboro, Maureen Aniedobe, Sunday Mogaji, Stephanie Ogechi Nwaiwu, Ibukun Ruth Akande, Rosemary Ajuma Audu, Babatunde Lawal Salako

**Affiliations:** 1 Center for Human Virology and Genomics, Nigerian Institute of Medical Research, Yaba, Lagos, Nigeria; 2 Clinical Science Department, Nigerian Institute of Medical Research, Yaba, Lagos, Nigeria; 3 Monitoring and Evaluation Unit, Nigerian Institute of Medical Research, Yaba, Lagos, Nigeria; 4 Federal Medical Center, Ebute-Metta, Lagos Nigeria; 5 Paelon Memorial Medical Center, Victoria Island, Lagos, Nigeria; 6 First Cardiology Consultants, Ikoyi, Lagos, Nigeria; 7 Body Soul and Spirit Project, Department of Community Health and Primary Care College of Medicine, University of Lagos, Lagos, Nigeria; 8 Clinical Diagnostic Department, Nigerian Institute of Medical Research, Yaba, Lagos, Nigeria; World Health Organization, SOUTH SUDAN

## Abstract

Healthcare workers (HCWs) are disproportionately infected with SARS-CoV-2 when compared to members of the general public; estimating the seroprevalence of SARS-CoV-2 antibody and SARS-CoV-2 infection rate among HCWs is therefore crucial. This study was carried out in four health facilities in Lagos Nigeria to determine the prevalence of IgG antibodies (seroprevalence) and SARS-CoV-2 active infection rate via a positive rtPCR result, the cross-sectional study was conducted between December 2020 and July 2021. Nasopharyngeal and blood samples were collected from HCWs and screened for SARS-CoV-2 infection using the rtPCR technique and antibody using the Abbott anti-SARS-CoV-2 IgG CMIA assay, respectively. Demographic and occupational exposures data were obtained and analysed using descriptive and inferential statistics, variables significant via inferential statistics were subjected to a multivariate analysis. A total of 413 participants were enrolled, with a mean age in years of 38.4±11.0. The seroprevalence was 30.9% (115/372) while 63/395 (15.9%) were actively infected with the virus. HCWs whose job role had direct contact with patients had a higher percentage of SARS-CoV-2 infection when compared with those not in direct contact, also being a health care worker was significantly associated with getting a positive COVID-19 PCR result. In conclusion the SARS-CoV-2 seroprevalence seen in this study was higher than national serosurvey estimates indicating HCWs are at higher risk of COVID-19 infection when compared to the general public. Vaccination and effective implementation of infection control measures are important to protect HCWs.

## Introduction

The novel coronavirus SARS-CoV-2, the causative agent of the COVID-19 pandemic has emerged to be a pre-eminent global challenge with about 598 million persons infected worldwide as of August 2022 [1]. Health care workers (HCWs) are usually first responders to health emergencies and have been reported to be disproportionately infected with SARS-CoV-2 compared to the general population; this is due mainly to occupational exposure to the patients being attended and infectious materials generated such as droplets, aerosols, and contaminated surfaces all of which typically are found in healthcare settings [2, 3]. Reports from WHO (2021), estimates that about 180 000 health care workers may have died from COVID-19 within the first 15 months of the pandemic [4]. Consequently, HCWs have potential for increased exposure to SARS-CoV-2 and the highly contagious nature of the virus leads to increased risk of HCWs contracting the disease [5]. Other occupational characteristics such as the role of HCW i.e. job description, setting, Personal Protective Equipment (PPE) availability, usage and proximity to infectious agents have been related to increased risk and hence seropositivity [2, 6].

Despite the association of these occupational features to seroprevalence, some reports have queried if these translate into a significantly higher risk of infection among HCWs relative to the risk of community transmission, and if so, which HCWs are most at risk [2, 7, 8].

Though vaccination has offered hope towards stemming the tide of the pandemic, HCWs remain at risk in localities where vaccines are not widely available or vaccine uptake is low [9]. Vaccine coverage in Nigeria is low, as at August 2022 only about 27,700,000 persons had been fully vaccinated out of a population of approximately 216,000,000 persons meaning the HCWs are at increased risk of infection compared to countries with wider vaccine coverage and acceptability [10, 11]. All the factors that influence the course of pandemic control make estimating the seroprevalence of SARS-CoV-2 antibody and SARS-CoV-2 infection rate among health care workers (HCWs) very important. Testing for IgG antibodies SARS-CoV-2 can detect past infection and keep accurate estimates of the seroprevalence of SARS-CoV-2 during a pandemic [12]. Estimates of seroprevalence in HCWs have been reported in countries around the world and seroprevalence levels differ by region and stage of the pandemic [12, 13].

This knowledge is critical for the determination of useful metrics and indices that will influence the understanding of the natural history of the disease, planning of public health response, and evaluation of intervention measures targeted at control and mitigation of the disease as the pandemic unfolds [12, 13]. Although many SARS-CoV-2 serological surveys have been performed among HCWs in industrialized countries, few have reported about HCWs in sub-Saharan African countries [8, 14].

Bridging this data gap concerning exposure and infectivity among HCWs in Lagos Nigeria is particularly important, considering Lagos is the epicenter of the COVID-19 pandemic in Nigeria, and has the sixth highest infectivity rate in Sub-Saharan Africa [11, 15]. Information on seroprevalence among HCWs will reveal individuals who have been exposed to the disease and the nature of the immune response that they have developed.

The PCR positivity rate will determine the degree of active infection spread among different categories of health workers. To address the knowledge gaps identified above, a study was conducted in four health facilities in Lagos Nigeria, that responded actively in controlling the pandemic through active testing and management of infected individuals. The goal was to assess the seroprevalence of anti-SARS-CoV-2 IgG antibodies, SARS-CoV-2 infection positivity rate, and the rate of infection in HCWs in direct contact with patients versus HCWs not in direct contact in these facilities.

## Materials and methods

### Ethical approval

The study proposal and protocol were approved by the Ethics Committee of the Nigerian Institute of Medical Research (NIMR) with reference number IRB/21/005.

### Study design

This is a cross-sectional study conducted between December 2020 and July 2021. Study participants were from four health facilities in Lagos Nigeria; consisting of two government-based (i.e., public) facilities as they were major referral centers for COVID-19 patient testing in Lagos state and two randomly selected private hospitals, which were listed and approved for COVID-19 care by the state government. Most times, private hospitals in Lagos are smaller when compared to public hospitals, hence have a lower number of HCWs. The institutions were: (i) NIMR) (ii) Federal Medical Centre (FMC), Ebute-Metta (iii) First Cardiology Hospital (FCH) and (iv) Paelon Memorial Medical Centre (PMMC).

NIMR is an apex medical research institute in the country, an agency of the Federal Ministry of Health (FMOH), at the forefront of COVID-19 disease response in the country. NIMR responded actively in controlling the pandemic through active laboratory testing and implementation of a modified drive-through sampling laboratory service- the first of its kind in Nigeria [3]. Federal Medical Centre Ebute-Metta, First Cardiology Hospital and Paelon Memorial Medical Centre were involved in actively controlling the pandemic through the management of infected individuals.

After a written informed consent was obtained, samples were taken from the HCWs at their respective places of work, and all laboratory analysis was done at the Center for Human Virology and Genomics (CHVG), a national reference laboratory located within NIMR. The laboratory has ISO 15189 accreditation and is listed as a World Health Organization (WHO) pre-qualification laboratory. Serological testing was done to assess the prevalence of SARS-CoV-2 IgG antibodies among HCWs and termed seroprevalence and SARS-CoV-2 molecular testing was done by rtPCR for COVID-19 positivity rate among the participants.

### Study population

The study population was made up of 413 consenting participants; whom were HCWs and other personnel above 18 years of age, involved in COVID-19 response in the selected health facilities listed above. In this study, HCWs are defined as “all staff involved in the direct or indirect provision of care to COVID-19 patients” and further included allied and auxiliary health workers such as cleaners, technicians, admission clerks, phlebotomists, researchers, scientists, etc. as defined by WHO [16]. Health workers in direct contact with patients were those whose role directly provided care and had contact with patients such as clinicians, nurses, and phlebotomists, any health worker who had no direct contact were classified as HCWs not in direct contact with patients while students, interns, and administrative staff were classified as others. A pre-designed case reporting form (CRF) was used to retrieve socio-demographic and clinical variables from all consenting consecutive participants, thereafter blood samples, oro-pharyngeal and nasopharyngeal swab samples were collected from respondents. Blood samples were collected prior to vaccination of the HCWs recruited for this study.

### Specimen collection for serological study

Ten milliliters of venous blood was obtained from each participant into plain vacutainer bottles. The samples were centrifuged at 4,000 rpm for 10 minutes and the serum was collected into clean cryovials. The serum samples were stored at -20°C until use. The sample collection took place in each of the medical facilities of the participating sites.

### Laboratory testing of SARS-CoV-2 antibodies

#### Serological antibody evaluation

Serum samples were screened for the presence of SARS-COV-2 antibody using the Chemiluminescent Microparticle Immunoassay (CMIA) technique by Abbott anti-SARS-CoV-2 IgG CMIA assay. The assay targets the nucleocapsid protein of SARS-CoV-2 virus, and assay results were interpreted as either positive or negative. The assay had been previously validated extensively on a SARS-CoV-2 characterized panel and results published before use in this study [13].

#### Sample collection for RT-PCR

Oropharyngeal (OP) and nasopharyngeal (NP) swabs were collected by inserting swabs to the posterior end of the nasal and throat regions, respectively, swirling the area for 10 seconds and immediately immersing swabs into the same tube of 2 ml of Viral Transport Medium (VTM). All samples were transported in a cold chain (2–8°C) to the Centre for Human Virology and Genomics (CHVG) of NIMR for laboratory analysis.

#### Testing of samples by RT-PCR

Viral RNA was manually extracted from oral and nasal swabs using the QIAamp viral RNA Mini Kit (Qiagen, Hilden, Germany), under a biosafety level 2 laminar flow cabinet. The viral RNA was subsequently amplified and detected using the Quant Studio 3 real-time PCR (Thermo Fisher Scientific Inc, USA) and the BGI real-time fluorescent RT-PCR kit (BGI, Shenzhen, China) according to the manufacturer’s instructions. The BGI assay detects the SARS-CoV-2 open reading frame 1 (ORF1) region, with an internal control detecting a human housekeeping gene. The internal control verifies that sampling was collected properly and checks for false negative or inhibition. Assay validation included ensuring curves are S-shaped, no cycle threshold (CT) values for the negative control, and both targets detected for the positive control with CT ≤ 32. All samples tested with internal control detected at CT ≤ 32 were accepted as valid. The assay has a limit of detection of 100 RNA copies/mL [17].

#### Data collection and statistical analysis

Data was retrieved from the CRFs and entered into NIMR REDCap platform. Statistical analysis was done with SPSS version 27. Univariate analysis was used to summarize the study data and presented in frequency tables and percentages, and other descriptive statistics such as mean and standard deviation were also done. Prevalence was calculated as number of participants with positive results divided by total number of participants who took the test and multiplied by 100. Continuous variables such as age and body mass index (BMI) were grouped categorically. Bivariate analysis was carried out using Pearson’s Chi-square to test for statistically significant association between the study outcomes (COVID-19 PCR test results and serology test results for COVID-19 antigen) and categorical variables (participants’ characteristics).

Associations were considered statistically significant if the p-value was less than or equal to 0.05 (p≤0.05). Variables that were statistically significant were subsequently subjected to multivariate analysis, using standard binary logistic regression, by adding each of the independent variable found to be significant (Job description) from the chi-square analysis to the logistic regression model. Binary logistic regression is used to predict the odds of having a positive PCR test result based on the independent variables. The dependent variables (serological test result and PCR test result) were dichotomous and were mutually exclusive. Indeterminate results were reclassified as negative for the analysis.

## Results

Table 1 shows the characteristics of participants in the study population. The mean age of participants was 38.4±11.0 years, and the age group 35–50 years had the highest proportion (40.9%). The participants were mostly female (55%), and HCWs who were not in direct contact with patients (48.7%). The proportion of participants who tested positive for the COVID-19 PCR test was 15.3%, 62% of the participants had a negative serological test, while 27.8% tested positive. NIMR made up 74.1% of the study population.

**Table 1:** Socio- demographic characteristics of participants.

Variables		Frequency (n = 413)	Percentage (100%)
**Age (Years)**			
	18–34	162	39.2
	35–50	169	40.9
	>50	68	16.5
	Missing	14	3.4
	Mean±SD	38.4±11.0	
	Total	413	100.0
**Sex**			
	Female	228	55.0
	Male	184	43.8
	Missing	1	0.2
	Total	413	100.0
**Job description**			
	Heath workers in direct contact with patients	143	34.6
	Health workers not in direct contact with patients	201	48.7
	Others	17	4.1
	Missing	52	12.6
	Total	413	100.0
**BMI(Kg/m^2) n = 212**			
	<18.5	14	3.4
	18.5–24.5	77	18.6
	25–29.9	68	16.5
	30–39.9	39	9.4
	>40	14	3.4
	Total	212	51.3
	Missing	201	48.7
	Total	413	100.0
**COVID-19 PCR Results**			
	Indeterminate	15	3.6
	Missing	18	4.4
	Negative	317	76.8
	Positive	63	15.3
	Total	413	100.0
**Serological IgG Test (ABBOTT)**			
	Missing	41	9.9
	Negative	257	62.2
	Positive	115	27.8
	Total	413	100.0
**Institutions**			
	First Cardiology	26	6.3
	FMC	53	12.8
	NIMR	306	74.1
	PAELON	28	6.8
	Total	413	100

Using the gender category (N = 412), active SARS-CoV_2 infection rate was = (63/63+332) *100 = 15.95%, while the Seroprevalence of IgG antibodies to SARS − CoV– 2 was 30.91% (115/115+257) *100 = 30.91%. This indicates that 15.95% of the participants who got a test had COVID-19 infection. The seroprevalence result indicates that 30.91% of the participants who got a test actually had antibodies to SARS − CoV − 2. IgG antibodies were present in 65 (28.5%) female and 50 male (27.2%) participants. IgG antibodies were also present in all the categories of occupation with the majority (30.3%) being Health workers not in direct contact with patients and the least (5.9%) being other categories of the participants such as IT students and interns. The age group >50 had the highest seroprevalence (IgG antibodies) at 32.4% while the 18–30 age group had the lowest at 24.6%. (Table 2).

**Table 2:** Description of COVID-19 PCR and SARS-CoV-2 serological tests results stratified by different participants’ demographics.

		COVID-19 PCR RESULTS COBAS		SARS-CoV-2 SEROLOGICAL TEST FINAL RESULT(ABBOTT)	
Variables	Total tested * (%)	Negative** (%)	Positive** (%)	Negative** (%)	Positive** (%)
**Age (Years) (N = 399)**					
18–30	122 (30.6)	98 (80.3)	20 (16.4)	83(68.0)	30 (24.6)
31–50	209 (52.4)	165 (78.9)	35 (16.7)	129 (61.7)	57 (27.3)
>50	68 (17.0)	58 (85.3)	6 (8.8)	42 (61.8)	22 (32.4)
**Gender (N = 412)**					
Female	228 (55.3)	179 (78.5)	39 (17.1)	144 (63.2)	65 (28.5)
Male	184 (44.7)	153 (83.2)	24 (13.0)	113 (61.0)	50 (27.2)
**Job description (N = 361)**					
Health workers in direct contact	143 (39.6)	112 (78.3)	24 (16.8)	85 (59.4)	36 (25.2)
Health workers not in direct contact	201 (55.7)	179 (89.1)	26 (12.9)	128 (63.7)	61 (30.3)
▪Others	17 (4.7)	12 (70.5)	2 (11.8)	16 (94.1)	1 (5.9)

▪Others are workers such as interns and students who are not healthcare workers

* Column percentage

** Row percentage

Table 3 below shows the job description to be statistically significant when stratified by COVID-19 PCR test results (p-value of 0.03). However, there was no significant association between the age, gender, and BMI of participants to COVID-19 positivity (p = 0.45; 0.48; 0.28).

**Table 3:** Comparison between participant’s demography and COVID-19 PCR results.

Variables	Negative	Positive	Total	p-value*
**Age (Years)**				0.45
18–34	128	29	157	
35–50	136	26	162	
>50	57	6	63	
**Total**	**321**	**61**	**382**	** **
**Gender**				0.48
Female	179	39	218	
Male	153	24	177	
**Total**	**332**	**63**	**395**	** **
**Job description**				**0.03**
Health workers in direct contact	112	24	136	
Health workers not in direct contact	170	26	196	
Others	12	2	14	
**Total**	**294**	**52**	**346**	** **
**BMI (Kg/m^2)**				0.28
<18.5	12	2	14	
18.5–24.5	63	8	71	
25–29.9	55	10	65	
30–39.9	35	1	39	
>40	10	3	14	
**Total**	**175**	**24**	**199**	** **

Participants’ characteristics stratified by COVID-19 PCR test. Statistically significant p-value highlighted in bold. Indeterminate test results that were neither positive nor negative were reclassified as negative while missing values were excluded from the analysis.

* Chi-squared test

Table 4 shows a statistically significant association between job description and IgG serology test (p-value = 0.003), while age, gender, and BMI have no statistically significant association with IgG seroprevalence (p-value = 0.73; 0.54; 0.97 respectively).

**Table 4:** Participants’ demographics versus SARS-CoV-2 Serological test IgG (ABBOTT).

Variables	Negative	Positive	Total	p-value*
**Age (years)**				0.73
18–34	83	30	113	
35–50	129	57	186	
>50	42	22	64	
**Total**	**254**	**109**	**363**	** **
**Gender**				0.57
Female	144	65	209	
Male	113	50	163	
**Total**	**257**	**115**	**372**	** **
**Job description**				**0.003**
Health workers in direct contact	85	36	121	
Health workers not in direct contact	128	61	189	
Others	16	1	17	
**Total**	**229**	**98**	**327**	** **
**BMI Kg/m^2**				0.94
<18.5	8	5	13	
18.5–24.5	50	17	67	
25–29.9	45	17	62	
30–39.9	23	11	34	
>40	8	5	13	
**Total**	**134**	**55**	**189**	** **

Participants’ characteristics stratified by serological test results. Statistically significant p-value highlighted in bold. Missing values were excluded from the analysis.* Chi-squared test

In Table 5, a binary logistic regression was performed to ascertain the effects of the job description on the likelihood that participants have positive serological and PCR test result. HCWs not in direct contact with patients are less likely to have positive PCR test results, but they have 1.13 times higher odds of having a positive serological test result than HCWs in direct contact with patients.

**Table 5:** Logistic regression showing the relationship between job description versus SARS-CoV-2 seroprevalence and SARS-CoV-2 positivity.

	SEROLOGY POSITIVE		95% CI FOR AOR		PCR COVID		95% CI FOR AOR	
Variables	p-value	AOR	Lower	Upper	p-value	AOR	Lower	Upper
**Job Description**								
Health workers in direct contact	0.14				0.55			
Health workers not in direct contact	0.64	1.13	0.69	1.85	0.27	0.71	0.39	1.31
Others	0.07	0.15	0.02	1.16	0.75	0.78	0.16	3.70

*AOR; adjusted odds ratio, C.I.; confidence interval. The variables associated with PCR and serological results in the bivariate analysis with p-value <0.05 were included in the multivariate model. Others are workers such as interns and students who are not healthcare workers.

## Discussion

This study reports the seroprevalence of HCWs in Lagos, the epicenter of the COVID-19 pandemic in Nigeria. Seroprevalence shows estimates of IgG antibodies of both asymptomatic and symptomatic persons infected with SARS-CoV-2, while a COVID-19 PCR test show results of current infectivity [16]. Past serosurveys in Lagos/Nigeria has focused mainly on the general populace, recruiting all age groups and job cadres not HCWs only while our study focuses mainly on adult HCWs [18]. Findings from our study reveal a seroprevalence of 30.91% and a 15.95% SARS-CoV-2 positivity infection rate among HCWs in Lagos Nigeria.

The health care sector is one of the hardest hit by the pandemic as HCWs have been reported to have increased probabilities of infection with SARS-CoV-2 than the general population; this was reflected in our study [2, 16]. The seroprevalence of SARS-CoV-2 observed in HCWs in our study at 30.91% was higher than estimates reported for the general population in Lagos state (23.3%) in the national serosurvey conducted in 2020, this was in conformity with various studies that observed increased seroprevalence in HCWs when compared to community seroprevalence [2, 18, 19]. Our study and the 2020 national serosurvey utilized similar serological testing techniques, which were well validated in-country prior to deployment in both studies [13, 18].

The seroprevalence among HCWs found in the study population in Lagos is however, lower than that of health workers in other Nigerian states; Ibadan, Oyo state at 45% and 37.21% in Niger state [20, 21]. The lower seroprevalence in Lagos may be due to the infection control measures implemented and the drive-through system and design of the sample collection site in NIMR where majority (74.1%) of the participants were located [3, 6].

The study results showed evidence of seroprevalence (IgG antibodies) across all cadres of HCWs. Health workers not in direct contact with patients and the age group > 50 years had the highest at 30.3% and 32.8%. There was also a significant association between the job description and positive IgGs seroprevalence (p = 0.003) other variables such as gender, BMI and age group were not significantly associated with seroprevalence. The association observed in this study was in concordance with previous works that observed that the health care workers’ job role was a risk factor for COVID -19 infection [7, 8, 16, 19].

SARS-CoV-2 positivity (COVID-19 PCR positive test results) was 15.9%, and was highest (16.8%) among HCWs in direct contact with patients than any other study categories, though this was not statistically significant in comparison to other groups. This finding was similar with other studies that confirmed HCWs in contact with patients with COVID-19 represent a high-risk group for SARS-CoV-2 infection [19–22]. This category of HCWs in direct contact with patients, had lower antibodies levels (seroprevalence of 25.2%) when compared to other groups; indicating wanantibodies or lesser prior exposure to SARS-CoV-2, this may be because they had to implement stringent infection control procedures as their job involved physical contact with infected patients.

When risk factors for SARS-CoV-2 positivity were examined, there was no significant association between gender and BMI, however being a HCW (job description) had significant association (p = 0.03) with getting a positive COVID-19 PCR test. This was consistent with other studies that identified the role of HCW as a risk factor for COVID-19 infection [2, 19, 22].

The study also examined the odds of having a positive SARS-CoV-2 serology and PCR test result among HCWs using a logistic regression model. Healthcare workers not in direct contact with patients were less likely to have active COVID-19 infection but had increased odds of a positive serological test result when compared to HCWs in direct contact with patients, but these findings did not reach statistical significance.

This study has some limitations. We utilized a qualitative antibody serological assay (Abbot), whose sensitivity decreases with the length of time post PCR, mainly because the targeted antibody wans and depletes over time; hence the seroprevalence measured here is at the time of test. The assay utilized had sensistivity of 71.3% when measured in the Nigerian population; hence seroprevalence results may be higher than observed in this study [13]. The study could not discriminate between community-acquired infections and those acquired from contact with patients so health workers in direct contact with patients may have acquired infection from the community.

In conclusion, a 30.91% seroprevalence to SARS-CoV-2 and PCR positivity of 15.9% was seen among healthcare workers in Lagos Nigeria. Our finding shows that being a health care worker is associated with a higher risk for SARS-CoV-2 infection. Vaccination and increased adherence to infection prevention and control practices, provision and effective use of personal protective equipment and quick detection and isolation of HCWs infected with SARS-CoV-2 are imperative to decrease the risk of SARS-CoV-2 infection among HCWs.
